# Systematic Understanding of Mechanism of Danggui Shaoyao San against Ischemic Stroke Using a Network Pharmacology Approach

**DOI:** 10.1155/2022/3747285

**Published:** 2022-01-05

**Authors:** Sijie Li, Yong Yang, Wei Zhang, Haiyan Li, Wantong Yu, Chen Gao, Jiali Xu, Wenbo Zhao, Changhong Ren

**Affiliations:** ^1^Beijing Key Laboratory of Hypoxia Translational Medicine, Xuanwu Hospital, Capital Medical University, Beijing 100053, China; ^2^Center of Stroke, Beijing Institute for Brain Disorder, Capital Medical University, Beijing 100053, China; ^3^School of Chinese Medicine, Beijing University of Chines Medicine, Beijing 100029, China

## Abstract

**Purpose:**

Danggui Shaoyao San (DSS) was developed to treat the ischemic stroke (IS) in patients and animal models. The purpose of this study was to explore its active compounds and demonstrate its mechanism against IS through network pharmacology, molecular docking, and animal experiment.

**Methods:**

All the components of DSS were retrieved from the pharmacology database of TCM system. The genes corresponding to the targets were retrieved using OMIM, CTD database, and TTD database. The herb-compound-target network was constructed by Cytoscape software. The target protein-protein interaction network was built using the STRING database. The core targets of DSS were analyzed by Gene Ontology (GO) and Kyoto Encyclopedia of Genes and Genomes (KEGG). Then, we achieved molecular docking between the hub proteins and the key active compounds. Finally, animal experiments were performed to verify the core targets. Triphenyltetrazolium chloride (TTC) staining was used to calculate the infarct size in mice. The protein expression was determined using the Western blot.

**Results:**

Compound-target network mainly contained 51 compounds and 315 corresponding targets. Key targets contained MAPK1, SRC, PIK3R1, HRAS, AKT1, RHOA, RAC1, HSP90AA1, and RXRA FN1. There were 417 GO items in GO enrichment analysis (*p* < 0.05) and 119 signaling pathways (*p* < 0.05) in KEGG, mainly including negative regulation of apoptosis, steroid hormone-mediated signaling pathway, neutrophil activation, cellular response to oxidative stress, and VEGF signaling pathway. MAPK1, SRC, and PIK3R1 docked with small molecule compounds. According to the Western blot, the expression of p-MAPK 1, p-AKT, and p-SRC was regulated by DSS.

**Conclusions:**

This study showed that DSS can treat IS through multiple targets and routes and provided new insights to explore the mechanisms of DSS against IS.

## 1. Introduction 

Stroke has the characteristics of high incidence, high recurrence rate, high fatality rate, high disability rate, and high economic burden, which seriously threatens human health and quality of life. Ischemic stroke (IS) accounts for 70% to 80% of all strokes, which results from sudden interruption of the blood supply to areas of the brain [1]. Neurological deficits such as disturbances to consciousness, cognitive and behavioral changes, paralysis, dysphagia, and aphasia are the major sequels of stroke. In China, stroke is the leading cause of death and contributes to a heavy disease burden [2]. The most effective treatment is currently recognized as intravenous thrombolysis and/or endovascular treatment, which prevent irreversible brain tissue damage by restoring blood flow reperfusion in time [3]. Although in situ clot retrieval can improve the recanalization rate of patients with ischemic stroke (mTICI of 2b/3) to 80–90%, it still exceeds 73% of patients who are left with functional disability or death (90 days of mRS ≥ 2) [4]. Therefore, it is necessary to explore neuroprotective drug to promote neurological function after IS and improve the prognosis of stroke [5].

Traditional Chinese medicine (TCM) has played an important part in maintaining health for thousands of years. In recent years, traditional Chinese drugs have been reported to possess protective effects on the nervous system, which has attracted the attention of researchers worldwide [6]. Danggui Shaoyao San (DSS) is a famous herbal formula composed of the following 6 raw herbs: Paeoniae Radix Alba (PRA), Atractylodes macrocephala Koidz. (AMK), Chuanxiong Rhizoma (CR), Angelicae Sinensis Radix (ASR), Poria cocos (Schw.) Wolf (PCW), and Alisma orientale (Sam.) Juz. (AOJ), which has been widely used in the treatment of various gynecological diseases [7, 8]. Recently, it was found that DSS is a potential therapeutic agent for the treatment of cognitive impairment and depression [9]. It is reported that DSS not only prevents cognitive impairment from Alzheimer's disease (AD) but also improves microcirculation in patients with asymptomatic cerebral infarction [10]. A number of studies indicate the potentials of DSS for improving neurological functions in poststroke treatment [11, 12]. DSS treatment also promotes focal angiogenesis and neurogenesis, attenuates neurological deficit scores, and improves memory functions in experimental rat models of cerebral ischemic-reperfusion injury [13, 14]. However, the underlying neuroprotection mechanisms of DSS against IS remain largely unknown.

Compared with the single-target therapeutic effect of chemical drugs, TCM compound ingredients have the overall therapeutic effect, which is usually modulated through various pathways and targets [15]. System biology, such as network pharmacology, contributes to reveal the biological networks in which drugs work. The integration strategy of network biology and multidirectional pharmacology is conducive to expand the available drug target space and is expected to enhance therapeutic efficacy, elevate clinical trial success rate, and decrease drug discovery costs [16]. In recent years, it has been well applied for drug discovery, especially in the field of research and development of Chinese medicine formulae [17].

**Figure 1:**
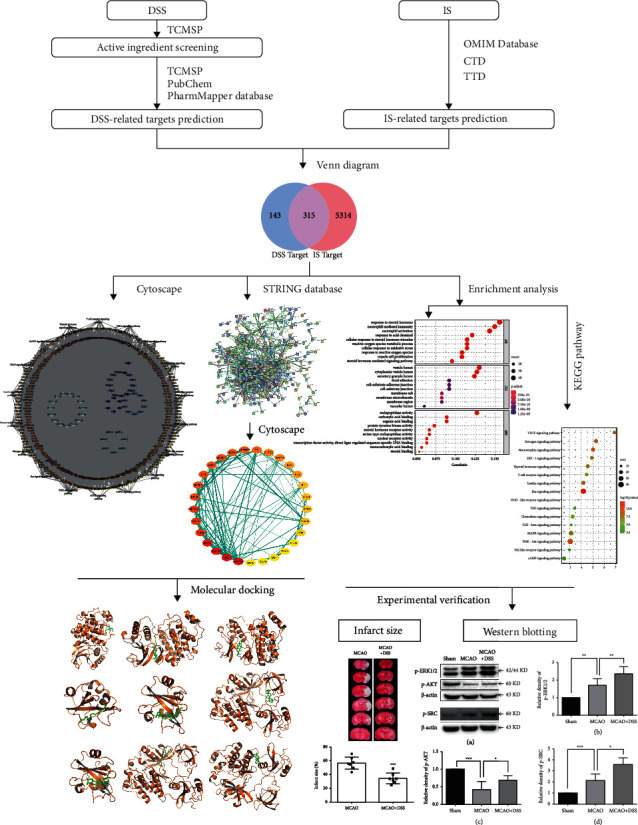
Flowchart of this study.

According to the description above, DSS is an ideal TCM in the application of treatment to IS. However, the underlying pharmacological mechanisms of DSS on IS treatment remain unclear. In this study, we used the emerging traditional Chinese medicine network pharmacology method to predict the targets of DSS and systematically predict the mechanism of action of DSS in IS. An outline of the method is shown in Figure 1. Our results offered the systematic mechanism of DSS in the treatment of IS and clearly clarified the synergy mechanism of DSS multicomponent and multi-target.

## 2. Materials and Methods

### 2.1. DSS Ingredient Collection and Target Gene Prediction

The traditional Chinese medicine systems pharmacology database and analysis platform (TCMSP, https://tcmsp-e.com/) was used for the active ingredient screening [18]. The names of herbs were used as the keywords to retrieve all components. We filtered active compounds by setting the pharmacokinetic index that the oral bioavailability (OB) was greater than 30% and the drug-like (DL) index was >0.18. Target genes were predicted using TCMSP, the PubChem website (pubchem.ncbi.nlm.nih.gov), and the PharmMapper database (http://www.lilabecust.cn/pharmmapper) after identifying the active ingredients [19].

### 2.2. Acquisition of Potential Therapeutic Targets for DSS Anti-IS

Online Mendelian Inheritance in Man (OMIM, http://omim.org) database, Comparative Toxicogenomics Database (CTD, http://ctdbase.org/) [20], and Therapeutic Target Database (TTD, http://db.idrblab.net/ttd/) were selected to obtain IS-related target gene [21]. All the databases used “ischemic stroke” as the keyword. The potential therapeutic targets of DSS in the treatment of IS were derived from the overlapping targets of DSS-related targets and IS-related targets. Subsequently, we used Cytoscape software (version 3.7.2) to construct a network diagram of “Drug active ingredient-target gene interaction network.” The topology analysis of the composition-target network was carried out using the function of “Network Analyzer” in the software.

### 2.3. Protein-Protein Interaction (PPI) Analysis

The obtained intersection targets were inputted to the STRING database (http://string-db.org/cgi/inpup.pl) with the species set to “*Homo sapiens*” to construct a protein-protein interaction (PPI) network [22]. The minimum required interaction score was set to “highest confidence (0.900),” and the hide disconnected nodes were set. Then, we obtained the PPI network. Next, the interaction files were downloaded and imported into Cytoscape software (version 3.7.2) to visualize the PPI network and analyze the topological value [23]. Then, the targets with the degree and betweenness centrality (BC), which were greater than the average value, were recognized as the key targets of DSS for the treatment of IS.

### 2.4. Enrichment Analysis of Gene Ontology (GO) and Kyoto Encyclopedia of Genes and Genomes (KEGG)

The predicted targets were imported into the Database for Annotation, Visualization, and Integrated Discovery (DAVID database, https://david.ncifcrf.gov) for GO and KEGG enrichment analyses [24]. GO enrichment analysis includes biological process (BP), cellular component (CC), and molecular function (MF). The enrichment results of GO and KEGG were obtained by setting the FDR value, and the bubble map was made according to the enrichment results [25].

### 2.5. Molecular Docking

The top three key compounds analyzed by “Network Analyzer” were obtained in the TCMSP database in mol2 format, and the top three key target proteins in the PPI network were obtained in the PDB database (http://www.rcsb.org). The software AutoDock Vina was used to carry out molecular docking between the key active ingredients and the key targets [26]. The binding energy was used as the evaluation index to evaluate the docking results, and the results were displayed by the software PyMOL [27].

### 2.6. Animal Model and Drug Administration

All animal experiments were approved by the Institutional Animal Care and Use Committee of Capital Medical University (XW-20210228-1) and in accordance with the principles outlined in the National Institutes of Health Guide for the Care and Use of Laboratory Animals. Male C57/BL6 mice (21–23 g) were used. Transient focal ischemia was induced by right middle cerebral artery occlusion (MCAO) using the intraluminal vascular occlusion method as previously described [28]. In brief, the right common carotid artery and the right external carotid artery (ECA) were exposed. The ECA was then dissected distally, ligated, and coagulated. The right MCA was occluded using a heparinized intraluminal filament. After 60 min, the suture was withdrawn. During surgery, rectal temperature was maintained at 37 ± 0.5°C with a thermostat-controlled heating pad. Sham-operated mice underwent an identical surgery except that the MCA was not occluded. Sixty minutes after occlusion, the filament was removed and a laser speckle contrast imaging (PSI System, Perimed Inc.) was used to observe the local cerebral blood flow. The mice were randomly assigned to the sham, MCAO, and MCAO + DSS groups. The MCAO + DSS group was administered DSS (20 g/kg) via the intragastric route at the time of reperfusion.

### 2.7. Preparation of DSS

The materials of DSS were purchased from Tong Ren Tang Pharmaceutical Company (Beijing, China) and were then authenticated by Dr. Weipeng Yang in the China Academy of Chinese Medical Sciences. The DSS dilution was prepared as described previously [14]. In brief, the 6 raw herbs were mixed in their dry weight ratios of 3 : 16 : 3:8 : 4:4 (ASR : PRA : CR : AOJ: PCW : AMK). The mixture was soaked in distilled water (1 : 8 w/v) for 30 minutes at room temperature, boiled for 1.5 h, and the extract was filtered thereafter. The boiling and extraction procedures were repeated three times. The extracted filtrate was concentrated using a rotary evaporator, and the final concentration of the extract is 1 g/ml (equivalent to the dry weight of the raw materials). The DSS extract was then stored at 4°C.

### 2.8. Two-Dimensional Laser Speckle Imaging

**Figure 2:**
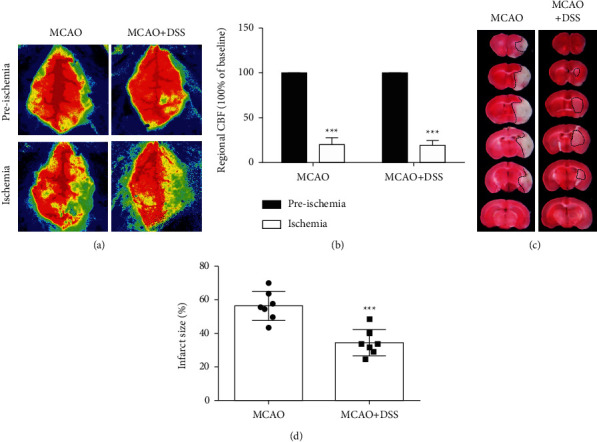
DSS treatment reduced infarct volume. (a) Regional cerebral blood flow (CBF) was monitored by the two-dimensional laser speckle imaging. (b) The blood flow ratio of two cerebral hemispheres (right/left), and a ratio less than 20% considered that the MCAO model is successful (*P* < 0.05). (c) Representative brain slices with infarcts stained by triphenyltetrazolium chloride from each group at 24 h after MCAO. (d) Quantification of infarct size at 24 h after MCAO. *N* = 7 per group. ^∗∗∗^*P* < 0.001, vs. MCAO group, by Student's *t*-test.

Regional cerebral blood flow (CBF) was monitored by the two-dimensional laser speckle imaging before ischemia and after the onset of ischemia (10 min after MCAO). We calculated the blood flow ratio of the two cerebral hemispheres (right/left), and at a ratio less than 20%, the MCAO model was considered successful (Figures 2(a)–2(b)).

### 2.9. Infarct Size Measurement

Infarct size was measured according to previous methods [28]. Twenty-four hours after surgery, the mice (*n* = 7 per group) were anesthetized with 1% pentobarbital sodium, and then, the brains were removed and sectioned coronally at 1-mm intervals to generate 6 slices. The slices were then incubated with 2% solution of 2, 3, 4-triphenyltetrazolium chloride (TTC). The infarct area and the corresponding contralateral area were measured by a blinded observer using the Image-Pro Plus software 5.0 (Rockville, MD, USA). Infarct size was calculated as a percentage of the size of contralateral hemisphere.

### 2.10. Western Blot

Tissue samples were collected from the ischemic hemisphere at 24 hours and 7 days, respectively, after reperfusion for the Western blot analysis. The samples at 24 hours after reperfusion were used for the detection of phosphorylated MAPK1 (p-ERK1/2) and phosphorylated RAC-alpha serine/threonine-protein kinase (p-AKT). The samples at 7 days after reperfusion were used for the detection of phosphorylated proto-oncogene tyrosine-protein kinase Src (p-SRC). Protein (40 *μ*g) was electrophoresed on 10% SDS polyacrylamide gels (Beijing Biotides Biotechnology Co., Ltd, Beijing, China) and then transferred to a polyvinylidene fluoride membrane (Millipore Corporation, USA). The membrane was probed with primary antibody: anti-p-ERK1/2 antibody (Cell Signaling; 1 : 1000 dilution), anti-p-AKT antibody (Cell Signaling; 1 : 1000 dilution), and anti-p-SRC antibody (Cell Signaling; 1 : 1000 dilution). The specific reaction was visualized through the use of a chemiluminescent substrate (GE Healthcare, UK) [29]. *β*-Actin was used to verify equal loading. The optical density of protein was measured using ImageJ software (NIH, Bethesda, MD, USA) according to the manufacturer's instructions (*n* = 7 per group).

### 2.11. Statistical Analysis

For two groups, the differences were analyzed for statistical significance by Student's *t*-test. For three or more groups, the differences were analyzed for statistical significance by the one-way ANOVA followed by Tukey's post hoc test. All the data were expressed as mean ± SD. The statistical analysis was performed with SPSS for Windows, version 21.0 (SPSS Inc.). The *p*-value <0.05 was considered significant.

## 3. Results

### 3.1. DSS Ingredient Collection and Target Gene Prediction

According to the two screening conditions of OB value and DL index, 54 active ingredients of DSS were obtained from TCMSP, including 13 ingredients in PRA, 7 ingredients in CR, 7 ingredients in AMK, 2 ingredients in ASR, 15 ingredients in PCW, and 10 ingredients in AOJ. ASR and PRA share one ingredient. PRA, AOJ, and CR share one ingredient (Table 1). Then, the target genes of 54 active ingredients were collected for target gene prediction in TCMSP and PubChem. A total of 14978 predicted targets were obtained. After removing the other species gene targets and duplicate values, 458 relevant human gene targets were obtained, including ASR (232), PRA (432), AOJ (377), CR(433), PCW (409), and AMK (359).

### 3.2. Drug Active Ingredient-Target Gene Interaction Network

**Figure 3:**
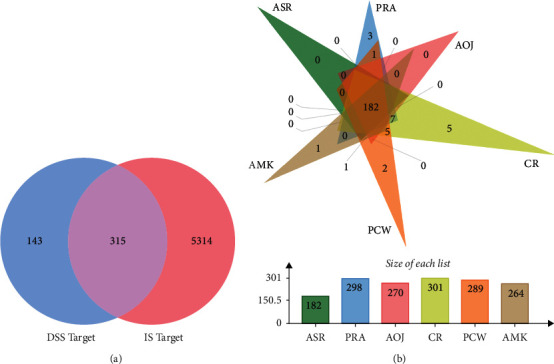
Identification of the drug-target interaction. (a) Venn diagram. (b) Drug-disease target analysis of traditional Chinese medicine. 182 represents the common targets of six Chinese medicines, and other figures indicate the unique targets of each Chinese medicine. PRA, *Paeoniae Radix Alba*. AMK, *Atractylodes macrocephala Koidz.*, CR, *Chuanxiong Rhizoma*. ASR, *Angelicae Sinensis Radix*. PCW, *Poria cocos* (Schw.) Wolf. AOJ, *Alisma orientale* (Sam.) Juz.

**Figure 4:**
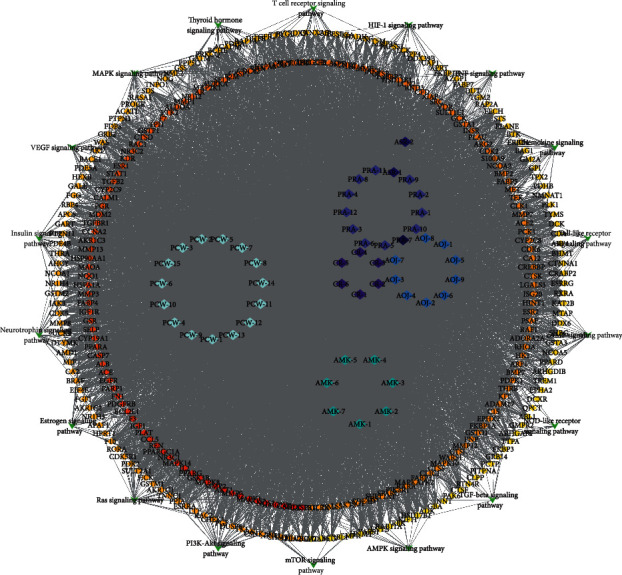
DSS prescription compositions of active ingredient-target pathway network. Rhombus represents the active ingredients of herbs contained in DSS. The degree value of a node represents the number of lines connected to the node in the network. The higher the degree value, the more important the node. The round nodes represent disease targets. The higher the degree of disease correlation, the redder the color is.

**Figure 5:**
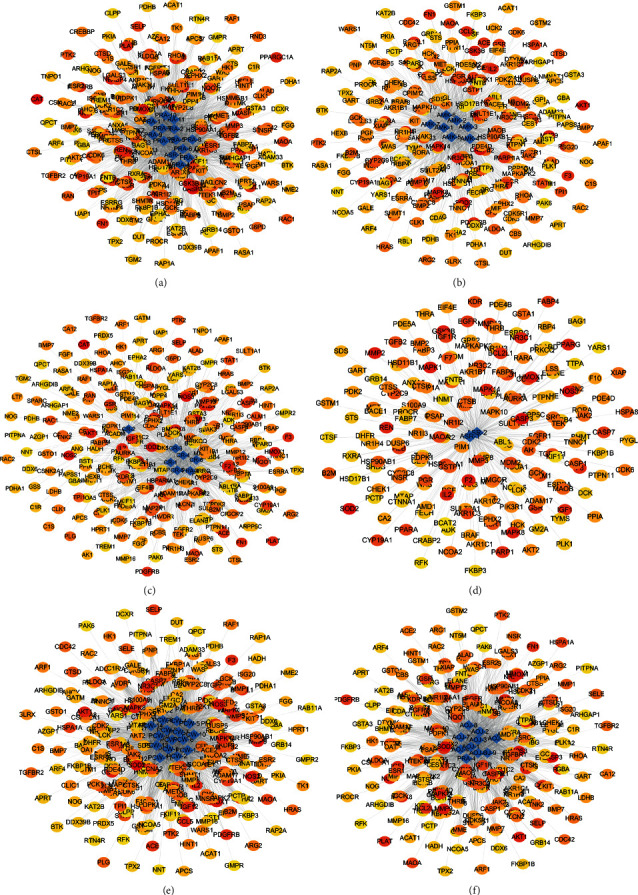
The network pharmacological diagram of a single herb. (a) PRA, *Paeoniae Radix Alba*. (b) AMK, *Atractylodes macrocephala Koidz.* (c) CR, *Chuanxiong Rhizoma*. (d) ASR, *Angelicae Sinensis Radix*. (e) PCW, *Poria cocos* (Schw.) Wolf. (f) AOJ, *Alisma orientale* (Sam.) Juz. Rhombus represents the active ingredients of herbs contained in DSS. The round nodes represent disease targets. The higher the degree of disease correlation, the redder the color is. The higher the degree value of the target gene to the herb, the closer it is to the herb component.

The overlapping targets of DSS-related targets and IS-related targets were considered as potential therapeutic targets for DSS anti-IS. As shown in Figure 1, a total of 315 intersection targets were obtained by screening drug ingredient targets and IS targets (Figure 3(a)). Six kinds of drugs have 182 common targets (Table 2) (Figure 3(b)). Drug active component-target gene interaction network contained 366 nodes (including 51 herb compound nodes and 315 target gene nodes) and 11146 edges. The degree value of a node represents the number of lines connected to the node in the network. The larger node means more importance. Among 315 target gene nodes, the higher the degree of disease correlation, the redder the color (Figure 4). In addition, in the network pharmacological map of a single herb, the higher the degree value of the target gene to the herb, the closer it is to the herb component (Figure 5). The topology analysis showed that the average degree value of each node in the network was 208.4706, and the average medium was 0.016732. There were 17 compound nodes with both degree and intermediate values above the mean (Table 3), suggesting that these compounds may be key compounds in the treatment of stroke.

### 3.3. Construction of the PPI Network and Core Target Screening

**Figure 6:**
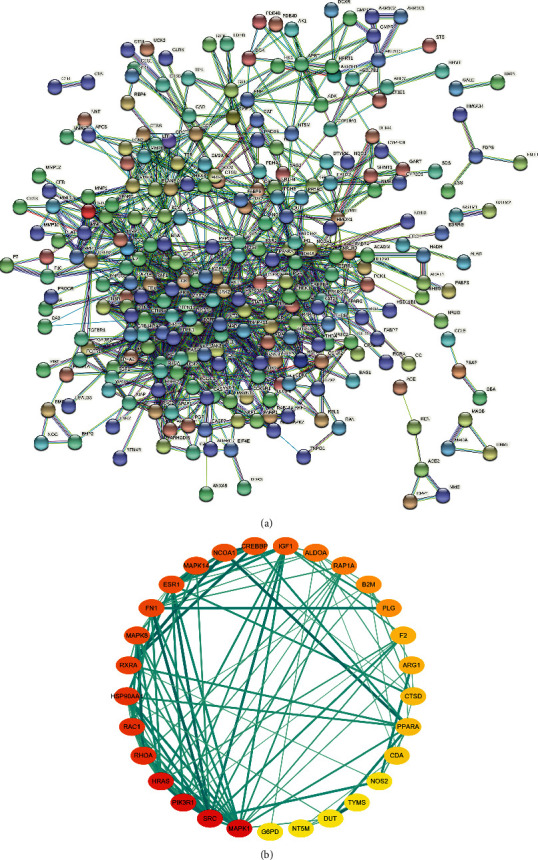
PPI network and top 30 hub genes for DSS anti-IS. (a) PPI network constructed with the STRING database. (b) Top 30 hub genes for DSS anti-IS were obtained using the degree and betweenness centrality algorithm. The node size and color indicate the degree value. The heavier the color, the greater the degree value and the wider the line and the heavier its color, indicating the closer the connection between the two proteins.

315 selected target genes were introduced into the STRING database to construct PPI network (Figure 6(a)). The PPI network contains 268 nodes and 1184 edges, indicating that 268 targets can interact with each other in the network, resulting in a total of 1184 interactions. After the analysis of topology parameters, the average node degree is 8.670412, and the average medium is 0.20796. There are 30 nodes with degree and intermediate values above the average, which were considered to be core targets of DSS in the treatment of IS (Figure 6(b), Table 4). According to the degree values, the top 10 core targets were identified as MAPK1, SRC, PIK3R1, HRAS, AKT1, RHOA, RAC1, HSP90AA1, RXRA, and FN1.

### 3.4. GO and KEGG Enrichment Analyses

**Figure 7:**
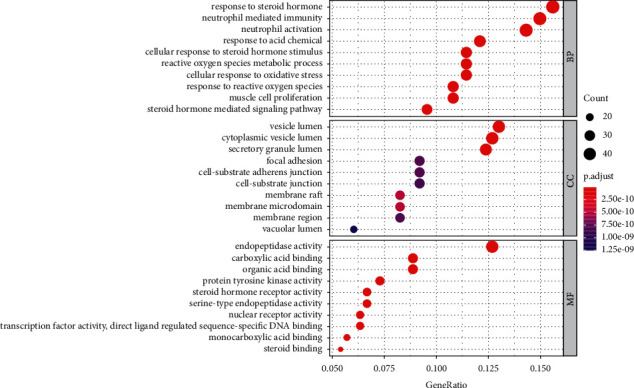
Functional classification of drug-disease targets by bioinformatic analysis. The biological process (BP), cellular component (CC), and molecular function (MF). Gene ratio refers to the ratio of enriched genes to all target genes, and counts refer to the number of the enriched genes.

315 targets screened by the DAVID database were used for GO functional enrichment analysis and KEGG signaling pathway enrichment analysis (FDR < 0.01). As a result, we obtained 417 GO terms, including 288 biological processes (BPs), 86 molecular functions (MFs), and 43 cellular components (CCs). BP mainly includes negative regulation of apoptosis, steroid hormone-mediated signaling pathway, neutrophil activation, and cellular response to oxidative stress. MF mainly includes the activity of steroid hormone receptor and protein tyrosine kinase. CC mainly includes cytoplasm and extracellular bodies. The top 10 screened BP, MF, and CC were selected as bubble charts through R language-related procedures (Figure 7).

**Figure 8:**
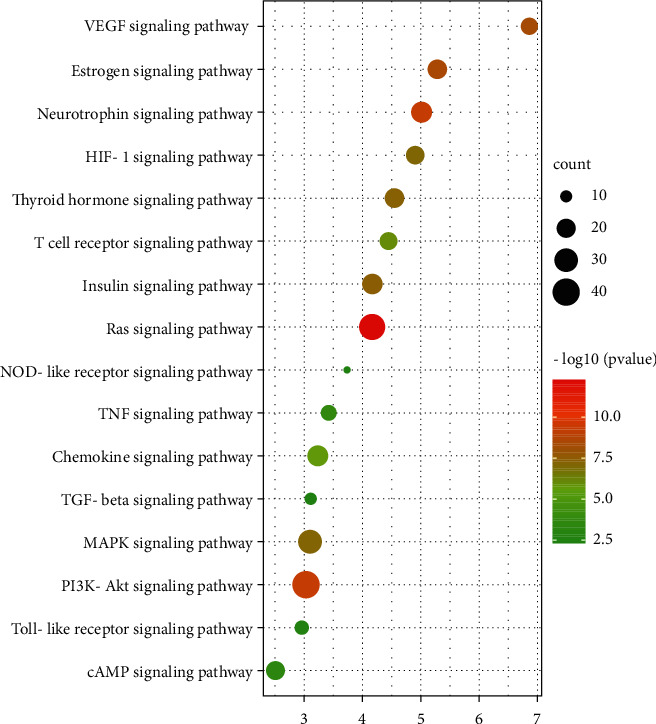
KEGG signaling pathway enrichment of screened genes. The bar chart showed the 16 pathways related to stroke. “Rich factor” represents the ratio of the number of target genes belonging to a pathway and the number of the annotated genes located in the pathway. A higher rich factor represents a higher level of enrichment. The size of the dot indicates the number of target genes in the pathway, and the color of the dot reflects the different *P* values.

The enrichment and screening of KEGG pathways resulted in 119 signaling pathways (FDR < 0.01) and 16 pathways related to stroke (Figure 8). The main pathway included VEGF signaling pathway, estrogen signaling pathway, neurotrophin signaling pathway, HIF-1 signaling pathway, and thyroid hormone signaling pathway.

### 3.5. Molecular Docking

**Figure 9:**
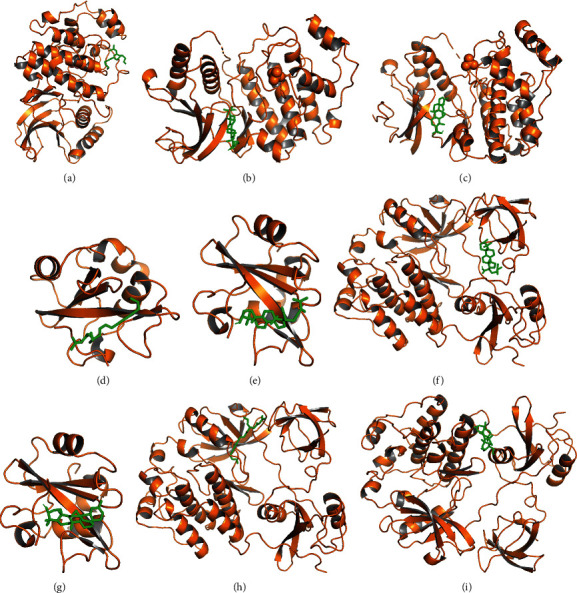
Docking results of the key ingredient and three hub target proteins. (a) 12-senecioyl-2E, 8E, 10E-atractylentriol and MAPK1; (b) eburicoic acid and MAPK1; (c) 11alpha,12alpha-epoxy-3beta-23-dihydroxy-30-nor-olean-20-en-28,12beta-olide and MAPK1; (d) 12-senecioyl-2E, 8E, 10E-atractylentriol and PI3K; (e) eburicoic acid and PI3K; (f) 11alpha,12alpha-epoxy-3beta-23-dihydroxy-30-nor-olean-20-en-28,12beta-olide and PI3K; (g) 12-senecioyl-2E, 8E, 10E-atractylentriol and SRC; (h) eburicoic acid and SRC; (i) 11alpha,12alpha-epoxy-3beta-23-dihydroxy-30-nor-olean-20-en-28,12beta-olide and SRC.

The top 3 key target proteins (MAPK1, PIK3R1, and SRC) in PPI network were selected to dock with the top 3 key active compounds (“11alpha, 12alpha-epoxy-3beta-23-dihydroxy-30-nor-olean-20-en-28,12beta-olide,” “eburicoic acid,” “12-senecioyl-2E, 8E, 10E-atractylentriol”). The PyMOL software was used to visualize the docking results of key active ingredients and core targets, as shown in Figure 9 and Table 5.

### 3.6. DSS Treatment Reduced Infarct Size

To confirm the neuroprotection of DSS in focal ischemic stroke, the MCAO mice were treated with DSS, and 24 hours later, the infarct size was detected by TTC stain. Compared with the MCAO group (56.47 ± 3.30), the infarct size was reduced by 39% in the MCAO + DSS group (34.49 ± 2.94) (*P*=0.0003) (Figure 2).

### 3.7. DSS Modulated the Expression of p-AKT, p-ERK1/2, and p-SRC

**Figure 10:**
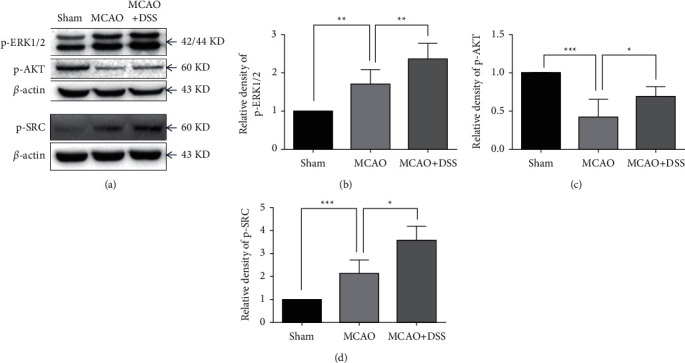
Expression of protein detected through the Western blot. (a) Representative Western blots of p-ERK1/2 and p-AKT at day 1 after MCAO surgery and the levels of p-SRC at day 7 after MCAO surgery. (b-d) Quantification of p-ERK1/2, p-AKT, and p-SRC, respectively. Values are mean ± SD. ^*∗*^*P* < 0.05, ^∗∗^*P* < 0.01, ^∗∗∗^*P* < 0.001. *N* = 7 per group.

To evaluate the performance of the core target screening approach used in this study, we selected the three major target proteins for analysis by the Western blot. The level of p-ERK1/2 was significantly increased in the MCAO mice (MCAO vs. sham, *p* < 0.01) and further significantly increased after LRIC treatment (MCAO + DSS vs. MCAO, *p* < 0.01) (Figure 10). Moreover, compared with the sham group, the expression of p-AKT in the MCAO group was decreased (*P* < 0.001), but that in the DSS group was significantly upregulated compared with the MCAO group (*p* < 0.05) (Figure 2). We also measured the expression levels of p-SRC at day 7 after MCAO surgery, finding that, when compared with the sham group, the levels of p-SRC in the MCAO mice were significantly increased (*P* < 0.001). DSS treatment for 7 days increased the levels of p-SRC compared with those found in the MCAO group (*P* < 0.05) (Figure 10).

## 4. Discussion

In this study, the anti-IS mechanism underlying the effect of DSS was explored using network pharmacology through data mining and subsequent computational modeling. It was noteworthy that our findings partially elucidate the complex anti-IS mechanism in the DSS, and they provide insight into the integrated understanding of the therapeutic efficacy and pharmaceutical activity of DSS. First, a total of 534 active ingredients were extracted from DSS. The possible targets of DSS were mined using the TCMSP database. PPI data were used to construct the core PPI network. Then, 30 candidate anti-IS targets of DSS were identified as the pivotal hub genes in the core PPI network according to the specific topological importance. In addition, GO and KEGG pathway analyses were carried out to demonstrate the candidate target biological significance after the pivotal hub genes were incorporated into ClueGO. Finally, we evaluate the neuroprotection role of DSS in IS stroke mice and detected the effect of DSS on the major drug targets in IS treatment using the Western blot.

### 4.1. DSS May Contribute to the Treatment of IS by Diminishing Apoptosis, Reducing Inflammation, and Oxidative Stress at Acute Stage

Cerebral ischemia-reperfusion injury induces a complex pathophysiological cascade that includes a wide range of aberrant cellular processes [30]. In the ischemic phase, reduced blood supply rapidly leads to failure of ionic gradients, excitotoxicity, and neuronal death. During the reperfusion phase, the return of oxygen contributes to oxidative stress, and the restoration of blood introduces factors that promote inflammation and edema, thereby further increasing the vulnerability of the affected tissue to death [30].

The increasing number of reports shows that apoptosis may contribute to a significant proportion of neuron death following IS [31]. Mechanistic studies have established that decreasing pro-apoptotic proteins or enhancing pro-survival proteins protects the brain after cerebral ischemia. We found that MAPK1 is the key target of DSS in the treatment of IS, and AKT1, GSK3, CASP3, and BCL2 are also among the targets. MAPK includes the extracellular signal-regulated kinase 1/2 (ERK1/2) subfamily. The phosphatidylinositol 3-kinase/Akt (PI3K/Akt) is an important signaling pathway involved in the regulation of cell apoptosis after stroke [32]. Many studies have shown that the activation of AKT and ERK1/2 can effectively inhibit apoptosis by regulating the expression of the anti-apoptosis gene Bcl-2 [33, 34]. Activated Akt promotes cell survival and suppresses apoptosis partly via inhibiting glycogen synthase kinase-3*β* (GSK3*β*), an apoptosis-related molecule [31]. Caspase-3 is considered to play an executing role at the final step of apoptosis-producing DNA fragmentation [31]. The report showed that DSS reduced the expression of caspase-3 and upregulated the expression of Bcl-2 at the MCAO rat model [11]. In this study, the Western blot results revealed that DSS significantly regulated the expression of p-ERK1/2, p-AKT, and Bcl-2. MAPK1 (ERK1/2) and PI3K, the two key targets, created 3D graphs of docking and analysis. Docking their crystal structures with the compounds, we showed that the two genes could be attached to the active pocket of the protein.

Inflammation after cerebral ischemia is a complicated pathological process starting from the activation of microglia, circulating leukocyte infiltrate (such as neutrophils, lymphocytes, and macrophages), and releasing of pro-inflammatory mediators mediated by ischemic cells and immune cells [30, 35]. Our GO analysis showed that DSS regulated neutrophil activation and neutrophil mediated immunity. Experimental models have shown that the inhibition of neutrophilic inflammatory mechanisms reduces neurodegeneration and improves functional outcome after cerebral ischemia [36]. Based on the KEGG pathway analysis, DSS influences TNF signaling pathway. TNF signaling pathway mediates a wide range of cellular processes including inflammation, proliferation, cell migration, apoptosis, and necrosis [37]. Studies confirm that the inhibition of TNF signaling pathway might be associated with reducing neuroinflammation in IS [38].

An imbalance between free radical production and antioxidant activity leads to oxidative stress, which is a major pathologic mechanism of secondary brain damage after cerebral ischemia [30]. Our GO analysis showed that the mechanism of DSS in the treatment of IS was related to cellular response to oxidative stress and reactive oxygen species metabolic process. Superoxide dismutase 2 (SOD2) and peroxisome proliferator-activated receptor alpha (PPARA) are the core targets of DSS in IS treatment. SOD2 is an enzymatic antioxidant that catalyzes the conversion of H2O2 and helps maintain the redox balance by diffusing the superoxide. Therapeutically increasing the levels of SOD2 could be an important treatment strategy in oxidative stress-induced pathology [39]. PPARA is a member of the PPAR nuclear receptor subfamily. Several studies have demonstrated that PPARA-mediated reduction in oxidative stress correlates with improved outcomes in rodent stroke models [40]. Recently, the study showed that DSS could attenuate oxidative stress against cerebral ischemic-reperfusion injury via SIRT1-dependent manner [11].

### 4.2. DSS May Contribute to the Treatment of IS by Increased Angiogenesis and Neurogenesis at Chronic Stage

Spontaneous neurogenesis and angiogenesis in the post-acute phase are highly coordinated responses and may contribute to the improvement in neurologic function after stroke. Accumulating experimental studies showed that promoting post-ischemic angiogenesis and neurogenesis can improve neurological function [41]. Ischemic stroke promotes neurogenesis by several growth factors including FGF-2, IGF-1, BDNF, VEGF, and chemokines including SDF-1 and MCP-1. Stroke-induced angiogenesis is similarly regulated by many factors most notably eNOS and CSE, VEGF/VEGFR2, and Ang-1/Tie2 [41]. KEGG enrichment analysis showed that VEGF signaling pathway and FGF signaling pathway were part of the ten major signaling pathways, suggesting that the mechanisms of DSS in the treatment of IS were related to the angiogenesis. SRC is the top three targets of DSS in the IS treatment. SRC is the downstream of VEGF to regulate angiogenesis. Our Western blot analysis confirmed that DSS significantly modulated the activity of SRC. We found that IGF-1, FGFR2, NOX3 (eNOS), MMP13, and EIF4E are the key targets of DSS in the treatment of IS. VEGF confers neuroprotection and promotes neurogenesis and cerebral angiogenesis after ischemic stroke [42]. VEGF signaling promotes endothelial nitric oxide synthase (eNOS) phosphorylation and is related to the angiogenesis [43]. Our previous study showed that DSS treatment promoted focal angiogenesis at 14 days after MCAO. Our previous study showed that DSS promotes angiogenesis and neurogenesis in rat following MCAO via upregulation of VEGF protein expression and increased eNOS activity [14]. The above studies have shown that DSS can not only inhibit apoptosis, inflammatory response, and oxidative stress in the acute phase of stroke but also improve neurological function by increasing neurogenesis and angiogenesis in the chronic recovery period. It suggests that DSS can be used in any period of stroke.

### 4.3. DSS May Contribute to the Treatment of IS by Modulate Estrogen Level

DSS is a famous herbal formulary from the Synopsis of the Golden Chamber, which was used to treat gynecological disease, especially female abdominal pain. At present, DSS is widely used in neurological diseases [7, 14]. Therefore, it is speculated that DSS might be able to regulate estrogen levels. Both GO and KEGG results showed that DSS responds to steroid hormone and regulates the estrogen signaling pathway. A number of evidence shows that the biological effects of estrogen extend beyond the gonads to other body systems, including the brain and behavior [44]. The results of preclinical studies have shown that estrogen has neuroprotective effects in various experimental models of IS [44]. Many different biological effects of estrogen are modulated by estrogen receptor (ESR), e.g., ESR1 and ESR2. Our result showed that ESR1 is a major target of DSS for IS treatment. The reports showed that estrogen reduces the ischemic oxidative damage via an ESR1-mediated inhibition of NADPH oxidase activation [45]. Dubal et al. showed that ESR1-/-mice have more severe brain damage after MCAO [46]. The above results might in part explain why DSS can treat both gynecological diseases and stroke.

Despite abundant new findings in this study, some limitations still exist. First, the corresponding experimental validation covered only a small number of mechanisms. Second, the compounds, targets, and pathways contained in these databases may not be exhaustive. In conclusion, the study, combined with network pharmacology, molecular docking, and animal experiments, offers the systematic mechanism of DSS in the treatment of IS and clearly clarifies the synergy mechanism of DSS multicomponent and multi-target. DSS may contribute to the treatment of IS by diminishing apoptosis, reducing inflammation and oxidative stress, increasing angiogenesis and neurogenesis, and modulating estrogen level. Our results indicate that DSS can not only reduce brain injury in the acute phase of stroke but also promote neurological recovery in the chronic phase of stroke.

## Figures and Tables

**Table 1:** Pharmaceutical ingredients.

Chinese name	Latin name	Molecule ID	Active ingredients	Ingredient code
Danggui	*Angelicae Sinensis Radix*	MOL000358	Beta-sitosterol	ASR-1
		MOL000449	Stigmasterol	ASR-2

Baishao	*Paeoniae Radix Alba*	MOL001921	Lactiflorin	PRA-1
		MOL001924	Paeoniflorin	PRA-2
		MOL000211	Mairin	PRA-3
		MOL000358	Beta-sitosterol	ASR-1
		MOL000359	Sitosterol	PRA-4
		MOL001930	Benzoyl paeoniflorin	PRA-5
		MOL001919	Palbinone	PRA-6
		MOL001925	Paeoniflorin	PRA-7
		MOL001910	11alpha,12alpha-epoxy-3beta-23-Dihydroxy-30-nor--olean-20-en-28,12beta-olide	PRA-8
		MOL001918	Paeoniflorin genome	PRA-9
		MOL001928	Albiflorin	PRA-10
		MOL000492	Cianidanol	PRA-11
		MOL000422	Kaempferol	PRA-12

Zexie	*Alisma Orientale* (Sam.) Juz.	MOL000856	Alisol C monoacetate	AOJ-1
		MOL000853	Alisol B	AOJ-2
		MOL000832	Alisol B 23-acetate	AOJ-3
		MOL000830	Alisol B	AOJ-4
		MOL000854	Alisol C	AOJ-5
		MOL000862	Alisol B 23-acetate	AOJ-6
		MOL000831	Alisol B monoacetate	AOJ-7
		MOL000849	16*β*-Methoxyalisol B monoacetate	AOJ-8
		MOL000359	Sitosterol	PRA-4
		MOL002464	1-Monolinolein	AOJ-9

Chuanxiong	*Chuanxiong Rhizoma*	MOL000359	Sitosterol	PRA-4
		MOL002157	Wallichilide	CR-1
		MOL000433	Folsaeure	CR-2
		MOL002135	Myricanone	CR-3
		MOL002140	Perlolyrine	CR-4
		MOL002151	Senkyunone	CR-5
		MOL001494	Mandenol	CR-6

Fuling	*Poria cocos* (Schw.) Wolf.	MOL000300	Dehydroeburicoic acid	PCW-1
		MOL000285	Polyporenic acid C	PCW-2
		MOL000280	Dehydrotumulosic acid	PCW-3
		MOL000273	16alpha-Hydroxydehydrotrametenolic acid	PCW-4
		MOL000283	Ergosterol peroxide	PCW-5
		MOL000276	7, 9 (11)-dehydropachymic acid	PCW-6
		MOL000289	Pachymic acid	PCW-7
		MOL000287	Eburicoic acid	PCW-8
		MOL000275	Trametenolic acid	PCW-9
		MOL000279	Cerevisterol	PCW-10
		MOL000290	Poricoic acid A	PCW-11
		MOL000296	Hederagenin	PCW-12
		MOL000292	Poricoic acid C	PCW-13
		MOL000291	Poricoic acid B	PCW-14
		MOL000282	Stellasterol	PCW-15

Baizhu	*Atractylodes macrocephala Koidz.*	MOL000033	(24S)-24-Propylcholesta-5-ene-3beta-ol	AMK-1
		MOL000028	Α-Amyrin	AMK-2
		MOL000021	14-Acetyl-12-senecioyl-2E, 8E, 10E-atractylentriol	AMK-3
		MOL000022	14-Acetyl-12-senecioyl-2E, 8Z, 10E-atractylentriol	AMK-4
		MOL000020	12-Senecioyl-2E, 8E, 10E-atractylentriol	AMK-5
		MOL000049	3*β*-Acetoxyatractylone	AMK-6
		MOL000072	8*β*-Ethoxy atractylenolide III	AMK-7

**Table 2:** Drug active ingredient-target gene (common targets of six drugs).

Gene	Gene ID
CASP3	836
SOD2	6648
NOS3	4846
HMOX1	3162
MAPK1	5594
F2	2147
MMP2	4313
GSK3B	2932
MAPK8	5599
IL-2	3558
PPARG	5468
NR3C1	2908
MAPK14	1432
REN	5972
F3	2152
IGF-1	3479
BCL2L1	598
PARP1	142
EGFR	1956
CASP7	840
ALB	213
CYP19A1	1588
PPARA	5465
GSR	2936
FABP4	2167
IGF-1R	3480
MMP3	4314
MAOA	4128
NQO1	1728
MMP13	4322
HSP90AA1	3320
CCNA2	890
AKR1C3	8644
MDM2	4193
TGFBR1	7046
CALM1	801
PGR	5241
TGFB2	7042
CYP2C9	1559
ESR1	2099
NR3C2	4306
KDR	3791
GSTP1	2950
F7	2155
AR	367
NR1I2	8856
AURKA	6790
MAP2K1	5604
B2M	567
CASP1	834
HSPA8	3312
MAOB	4129
FGFR1	2260
HSP90AB1	3326
HMGCR	3156
JAK2	3717
MME	4311
CTSB	1508
LCN2	3934
HSD11B1	3290
SRC	6714
XIAP	331
SULT1E1	6783
VDR	7421
GSTA1	2938
GCK	2645
INSR	3643
CDK2	1017
NCOA2	10499
S100A9	6280
FABP5	2171
BMP2	650
TEK	7010
MET	4233
CDK6	1021
CYP2C8	1558
CTSK	1513
PSAP	5660
THRB	7068
PDPK1	5170
ADAM17	6868
KIT	3815
EPHX2	2053
FKBP1A	2280
MMP12	4321
CTSS	1520
MAPK10	5602
FABP3	2170
RARA	5914
MAPKAPK2	9261
PIK3R1	5295
PDE4D	5144
LSS	4047
AKT2	208
PIM1	5292
AKR1B1	231
PLA2G2A	5320
NR1I3	9970
OAT	4942
DUSP6	1848
PGF	5228
CHEK1	1111
ESRRA	2101
PPIA	5478
TNNC1	7134
AKR1C1	1645
GSTM1	2944
HCK	3055
SULT2A1	6822
PDK2	5164
F10	2159
RORA	6095
NR1H3	10062
AKR1C2	1646
EIF4E	1977
BRAF	673
CA2	760
AMD1	262
MMP8	4317
JAK3	3718
NCOA1	8648
NR1H4	9971
THRA	7067
PDE4B	5142
PTPN11	5781
GART	2618
RBP4	5950
PDE5A	8654
BACE1	23621
WAS	7454
GRB2	2885
PTPN1	5770
PROCR	10544
SDS	10993
PPP5C	5536
PRKCQ	5588
BCHE	590
ACADM	34
DHFR	1719
FGFR2	2263
TTR	7276
PYGL	5836
GC	2638
ANXA5	308
CES1	1066
LCK	3932
DPP4	1803
FKBP1B	2281
FABP7	2173
FECH	2235
STS	412
ELANE	1991
BAG1	573
ERBB4	2066
GM2A	2760
TPX2	22974
PLK1	5347
TYMS	7298
DCK	1633
BHMT	635
CTNNA1	1495
CRABP2	1382
ESRRG	2104
RXRA	6256
MTAP	4507
SHBG	6462
NCOA5	57727
PPARD	5467
ABL1	25
TTPA	7274
FKBP3	2287
GRB14	2888
PCTP	58488
CTSF	8722
ADK	132
HSD17B1	3292
KIF11	3832
RFK	55312
FNTB	2342
YARS1	8565
HNMT	3176
BCAT2	587

**Table 3:** Key compounds. (The average degree value is 208.4706, and the average medium is 0.016732.)

Compound	Compound ID	Degree	Betweenness centrality
11alpha,12alpha-epoxy-3beta-23-Dihydroxy-30-nor-olean-20-en-28,12beta-olide	PRA-8	228	0.024789
Eburicoic acid	PCW-8	226	0.017975
12-Senecioyl-2E, 8E, 10E-atractylentriol	AMK-5	225	0.021314
Dehydroeburicoic acid	PCW-1	225	0.01739
14-Acetyl-12-senecioyl-2E, 8Z, 10E-atractylentriol	PRA-11	223	0.028611
Cianidanol	AMK-4	223	0.01698
Alisol C	AOJ-5	221	0.017782
16alpha-Hydroxydehydrotrametenolic acid	PCW-4	221	0.017585
Poricoic acid C	PCW-13	220	0.021088
Palbinone	PRA-6	219	0.024345
Cerevisterol	PCW-10	217	0.017087
Poricoic acid A	PCW-11	216	0.02044
Kaempferol	PRA-12	215	0.032249
Paeoniflorin	PRA-2	215	0.017069
Lactiflorin	PRA-1	214	0.016884
Poricoic acid B	PCW-14	213	0.021272
Myricanone	CR-3	209	0.041457

**Table 4:** Drug targets of IS. (The average degree value is 8.670412, and the average medium is 0.20796.)

Gene	Protein name	Degree	Betweenness centrality
MAPK1	Mitogen-activated protein kinase 1	53	0.126385
SRC	Proto-oncogene tyrosine-protein kinase Src	50	0.052449
PIK3R1		47	0.047873
HRAS	GTPase HRas	44	0.033552
AKT1	Phosphatidylinositol 3-kinase regulatory subunit alpha	40	0.109791
RHOA	Transforming protein RhoA	35	0.047081
RAC1	Ras-related C3 botulinum toxin substrate 1	34	0.0268
HSP90AA1	Heat shock protein HSP 90-alpha	33	0.059285
RXRA	Retinoic acid receptor alpha， RAR-alpha	30	0.067159
FN1	Fibronectin 1	29	0.023024
MAPK8	Mitogen-activated protein kinase 8	29	0.022235
ESR1	Estrogen receptor	28	0.091184
MAPK14	Mitogen-activated protein kinase 14	28	0.058202
NCOA1	Nuclear receptor coactivator 1	27	0.039326
CREBBP	CREB binding protein	26	0.03777
IGF-1	Insulin-like growth factor I	25	0.024056
ALDOA	Fructose-bisphosphate aldolase A	22	0.065989
RAP1A	Ras-related protein Rap-1A	21	0.026304
B2M	Beta-2-microglobulin	19	0.034377
PLG	Plasminogen	19	0.023626
F2	Prothrombin	16	0.028418
ARG1	Arginase-1	16	0.021575
CTSD	Cathepsin D	15	0.037545
PPARA	Peroxisome proliferator-activated receptor alpha	15	0.028961
CDA	Cytidine deaminase	14	0.059555
TYMS	Thymidylate synthase	11	0.037145
NOS3	Nitric oxide synthase 3	11	0.036816
DUT	Deoxyuridine triphosphatase	9	0.057749
NT5M	5′(3′)-Deoxyribonucleotidase, mitochondrial	9	0.048718
G6PD	Glucose-6-phosphate dehydrogenase	9	0.027099

**Table 5:** Binding energy between the key ingredients and target proteins.

NO.	Receptor protein	Molecule ID	Ligand component	Binding energy (kcal·mol-1)
1	MAPK1	MOL000020	12-Senecioyl-2E, 8E, 10E-atractylentriol	−5.99
2	MAPK1	MOL000287	Eburicoic acid	−8.64
3	MAPK1	MOL001910	11alpha,12alpha-epoxy-3beta-23-Dihydroxy-30-nor-olean-20-en-28,12beta-olide	−9.32
4	PIK3R1	MOL000020	12-Senecioyl-2E, 8E, 10E-atractylentriol	−6.66
5	PIK3R1	MOL000287	Eburicoic acid	−9.24
6	PIK3R1	MOL001910	11alpha,12alpha-epoxy-3beta-23-Dihydroxy-30-nor-olean-20-en-28,12beta-olide	−10.18
7	SRC	MOL000020	12-Senecioyl-2E, 8E, 10E-atractylentriol	−7.57
8	SRC	MOL000287	Eburicoic acid	−9.42
9	SRC	MOL001910	11alpha,12alpha-epoxy-3beta-23-Dihydroxy-30-nor-olean-20-en-28,12beta-olide	−9

## Data Availability

The data that support the findings of this study are available from the corresponding author upon reasonable request.
